# Characterization of *Staphylococcus aureus* isolated from milk samples of dairy cows in small holder farms of North-Western Ethiopia

**DOI:** 10.1186/s12917-018-1558-1

**Published:** 2018-08-23

**Authors:** S. A. Mekonnen, T. J. G. M. Lam, J. Hoekstra, V. P. M. G. Rutten, T. S. Tessema, E. M. Broens, A. E. Riesebos, M. P. Spaninks, G. Koop

**Affiliations:** 1 Department of Farm Animal Health, Faculty of Veterinary Medicine, Utrecht University, Yalelaan 7, 3584 CL Utrecht, The Netherlands; 2 Faculty of Veterinary Medicine, University of Gondar, P.O. Box 196, Gondar, Ethiopia; 3 GD Animal Health, P.O. Box 9, 7400 AA Deventer, The Netherlands; 4 Department of Infectious Diseases and Immunology, Faculty of Veterinary Medicine, Utrecht University, Yalelaan 1, 3584 CL Utrecht, The Netherlands; 5 Department of Veterinary Tropical Diseases, Faculty of Veterinary Science, University of Pretoria, Private Bag X04, Onderstepoort, Pretoria, 0110 South Africa; 6 Institute of Biotechnology, Addis Ababa University, P.O. Box 1176, Addis Ababa, Ethiopia

**Keywords:** *Staphylococcus aureus*, *Spa*, MLST, Virulence factor, Antimicrobial resistance, Mastitis, Ethiopia

## Abstract

**Background:**

*Staphylococcus aureus* is a contagious, opportunistic pathogen that causes clinical or subclinical mastitis in dairy cattle. The genetic background and antimicrobial resistance of isolates from Ethiopian dairy farms has not been studied. Therefore, the aim of this study was to characterize *S. aureus* from Ethiopian hand milked dairy cows, by *spa,* MLST and virulence factor typing, and by assessment of antimicrobial susceptibility. A total of 79 *S. aureus* isolates from intramammary infections was studied. A PCR was used to detect *lukM-lukF’* and *pvl* genes encoding the bovine and human associated bi-component leukocidins, and the toxic shock syndrome toxin gene-1 (*tst*). Antimicrobial susceptibility was determined using the broth microdilution method.

**Results:**

Twenty different *spa* types were identified, most isolates were t042 (58%), and the closely related t15786 (11%). The proportion of isolates positive for *lukM-lukF’*, *tst* and *pvl* was low at 0.04, 0.10 and 0.09 respectively, with *lukM-lukF’* often co-occurring with *tst*, but not with *pvl*. Methicillin-resistance was not found, but resistance to penicillin/ampicillin (86%) and tetracycline (54%) was very common.

**Conclusions:**

We found a high degree of relatedness among bovine *S. aureus* isolates in North-Western Ethiopia, suggesting contagious within and between farm transmission of strains that are often resistant to commonly used antimicrobials. This highlights the need for effective preventive measures that aim at limiting transmission of bacteria rather than using antimicrobials to control *S. aureus* mastitis in Ethiopia.

**Electronic supplementary material:**

The online version of this article (10.1186/s12917-018-1558-1) contains supplementary material, which is available to authorized users.

## Background

*Staphylococcus aureus* is a contagious pathogen that causes mastitis in dairy cattle [1, 2], and is an opportunistic pathogen in humans and many other animal species [3–5]. Also in Ethiopia, *S. aureus* is frequently isolated from cows with mastitis [6–8], and from milk or milk products [9].

In dairy cows, *S. aureus* can be isolated from milk as well as from different other body sites [10, 11]. Transmission of *S. aureus* intramammary infections (IMI) is believed to mainly occur during the milking process [12], but this has been poorly researched in situations where hand milking is common, as is the case in Ethiopia. Additionally, hand milking may introduce possibilities for transmission between farmers and their cattle. Typing of *S. aureus* isolates may give insight in their likely origin, because MLST and spa-types and virulence factors often are host-associated [13, 14]. Genetic similarity of isolates within and between farms suggests contagious transmission in the spread of bacteria [4] whereas a greater variety of genotypes within herds or regions is more suggestive of environmental pathogens [15]. Genotyping does not only give information on modes of transmission, but can also be used to identify virulence factors of the bacteria. Depending on their clonal lineage, *S. aureus* may carry the phage-encoded leukocidin genes *lukM-lukF’* [16–18]. LukMF’ is a potent toxin specifically killing bovine neutrophils [19] and likely to contribute to the clinical severity of mastitis [20]. The proportion of bovine *S. aureus* isolates encoding this toxin varies largely between countries [21], but to our knowledge this has not been estimated in isolates of IMI from African cattle.

Because the use of antimicrobial drugs, both in humans and animals, is poorly controlled, multidrug resistant *S. aureus* are frequently isolated from animals [6, 7] and humans in Ethiopia [22–24]. In *S. aureus* isolated from humans, there is a trend of increasing antimicrobial resistance (AMR) [25]. Describing AMR patterns in both humans and animals may contribute to the knowledge on the importance of the issue in Ethiopia and to a more prudent and effective antimicrobial use.

In smallholder dairy farming, effective control of *S. aureus* mastitis is important, because mastitis can have a substantial effect on family income. Since only a limited number of studies characterized bovine *S. aureus* isolated from Ethiopia [9, 26], the aim of the present study was to characterize *S. aureus* isolates from milk samples from hand milked dairy cows in North-Western Ethiopia by *spa* and MLST typing, virulence genes and antimicrobial susceptibility.

## Methods

### Isolate collection

A total of 135 phenotypically isolated *S. aureus* were available from an earlier cross-sectional study conducted from October 2014 to December 2016 in 167 urban and peri-urban smallholder dairy farms in the regions Gondar and Bahir Dar, in the North-West of Ethiopia [8]. Nine isolates were lost. Forty seven isolates were excluded from the study because data of isolates were lost (11), data mismatched (8), and genotypically non *S. aureus* (28). Seventy nine *S. aureus* isolates that had complete data records were available (two from clinical mastitis (CM), 53 from California mastitis test (CMT) positive and 24 from CMT negative quarters) for *spa* and MLST typing, virulence genes detection and antimicrobial susceptibility testing. The 79 isolates were derived from 60 dairy cows in 42 dairy herds and were kept in glycerol stocks at − 80 °C until further study.

### DNA extraction

Single colonies from an overnight culture grown on blood agar plates were suspended in 1 mL of distilled water, homogenized using Vortex and centrifuged for 1 min at 13,000 rpm. Supernatant was removed and the bacteria were re-suspended in 200 μL of distilled water, boiled for 10 min at 100 °C and centrifuged at 13,000 rpm for 1 min. The extracted DNA was diluted 1:10 in distilled water and stored at − 20 °C until further analysis.

### Virulence genes detection

Polymerase chain reactions were performed on 10 μL of the DNA of all 79 isolates to confirm the isolates were *S. aureus* (*femA)*, to test for presence of the *mecA* gene, encoding resistance to methicillin, and to amplify the polymorphic X region of the surface protein A (*spa*) gene. We also used PCR to detect *lukM-lukF’* and *pvl* genes encoding the bovine and human associated bi-component leukocidins, respectively, and the toxic shock syndrome toxin gene-1 (*tst*). Primers and protocols are summarized in Table 1. Amplifications were carried out in final volumes of 25 μL using a T100™ Thermal Cycler (Bio-Rad, USA). The presence of appropriate amplicon sizes was examined by electrophoresis of 10 μL PCR product on 1.5% agarose at 100 V for 40 min. PCR products were visualized with a molecular Imager Gel Doc XR+ Imaging system (Bio-Rad, USA).

**Table 1:** Sequences of primer sets with their corresponding PCR protocols and product sizes

Target gene	Primer sequence (5′-3′)	PCR-protocol	Amplicon size	Reference
*femA*	F: TGCCTTTACAGATAGCATGCCA	1	142 bp	[52]
	R: AGTAAGTAAGCAAGCTGCAATGACC			
*mecA*	F: GGCTATCGTGTCACAATCGTT	2	689 bp	[53]
	R: TCACCTTGTCCGTAACCTGA			
*spa*	F: AGACGATCCTTCGGTGAGC	3	Variable	[54]
	R: GCTTTTGCAATGTCATTTACTG			
*lukM*	F: TGAGTGGGTATGGCATGAAAGA	4	572 bp	[20]
	R: TGGACATTTTGTGTTACACCCC			
*lukF’*	F: ACTCAGGCTATACCAACCCA	1	425 bp	[20]
	R: CGAGCTACTCTGTCTGCCAC			
*pvl*	F: GCTGGACAAAACTTCTTGGAATAT	5	85 bp	[55]
	R: GATAGGACACCAATAAATTCTGGATTG			
*tst*	F: CAACATACTAGCGAAGGAACT	6	277 bp	[56]
	R: GATATGTGGATCCGTCATTCA			

^1^ 95 °C for 2 min, 35× (95 °C for 30 s, 59.5 °C for 30 s, 72 °C for 35 s), 72 °C for 5 min and 12 °C for ∞

^2^ 95 °C for 1.5 min, 30× (95 °C for 45 s, 55 °C for 30 s, 72 °C for 45 s), 72 °C for 5 min and 12 °C for ∞

^3^ 95 °C for 2 min, 30× (95 °C for 45 s, 60 °C for 45 s, 72 °C for 1:30 min), 72 °C for 5 min and 12 °C for ∞

^4^ 95 °C for 2 min, 33× (95 °C for 30 s, 58.2 °C for 30 s, 72 °C for 30 s), 72 °C for 5 min and 12 °C for ∞

^5^ 95 °C for 2 min, 35× (95 °C for 30 s, 55.5 °C for 30 s, 72 °C for 35 s), 72 °C for 5 min and 12 °C for ∞

^6^ 95 °C for 2 min, 35× (95 °C for 30 s, 59 °C for 30 s, 72 °C for 30 s), 72 °C for 5 min and 12 °C for ∞

### *Spa* and MLST typing

The product of the *spa* gene PCR was cleaned up from unbound primers and nucleotides using ExoSAP-IT PCR Cleanup Reagent (Affymetrix, USA) according to manufacturer’s instructions and was submitted for Sanger sequencing (Baseclear BV, The Netherlands). The *spa* types were assigned using BioNumerics version 7 (Applied Maths, Belgium), using the *spa*-typing plugin and Ridom SpaServer (http://www.spaserver.ridom.de). A minimum spanning tree (MST) was constructed in BioNumerics using the *spa* clustering method of the spa typing plugin. Repeat successions were aligned using a gap creation cost of 100%, gap extension cost of 50%, duplicate creation and duplicate extension costs of both 25% and a maximum duplication number of 3 repeats. The MST was built using the MST cluster method and the default cost matrix with a 1% grouping distance bin. To save costs, multilocus sequence typing (MLST) as described by Enright et al. [27] was performed on a selection of ten of the 79 isolates: on six t042 isolates, and on 4 isolates of *spa* types t273, t17184, t488 and t409. These ten isolates were selected aiming to optimally represent the various spa types and the two regions (Gondar and Bahir Dar). Sequence types were assigned using BioNumerics.

### Antimicrobial susceptibility testing

Antimicrobial susceptibility testing was performed by determining minimum inhibitory concentrations (MICs) using the MICRONAUT system (Merlin Diagnostika, Germany) in customized prepared 96-wells microtiter plates containing dilution series of 17 antimicrobials. Inoculum preparation, broth composition and incubation conditions were performed as recommended by the manufacturer. Reading of the plates after incubation was done with a photometer (Skan, Merlin Diagnostika, Germany). *Staphylococcus aureus* ATCC 29213 was used as quality control strain. Clinical breakpoints were used according to the Clinical and Laboratory Standards Institute (CLSI) standards [28, 29]. Veterinary breakpoints were used for ceftiofur (cattle), cephalotin (dogs) and enrofloxacin (dogs). Because veterinary breakpoints for several antimicrobials have not been described, human breakpoints were used for penicillin, ampicillin, tetracycline, clindamycin, erythromycin, trimethoprim sulfamethoxazole, rifampicin, amoxicillin/clavulanic acid, cefoxitin, chloramphenicol, gentamicin, kanamycin and neomycin. For fusidic acid, EUCAST-criteria were used (www.eucast.org), because no breakpoints were available in the CLSI standards.

## Results

### Genetic typing

**Fig. 1:**
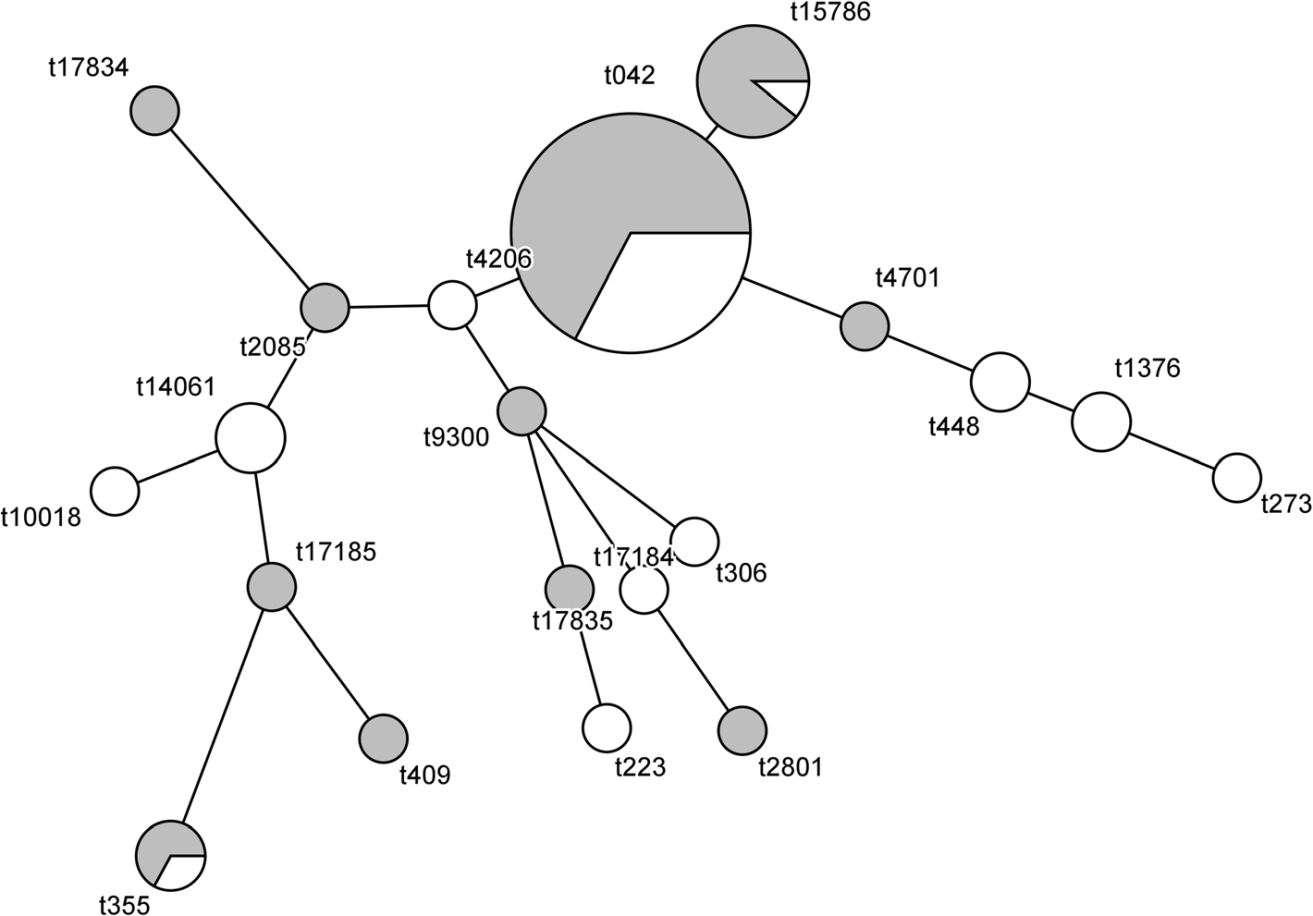
Minimum spanning tree based on *spa* types of 79 *S. aureus* isolates from Bahir Dar region (grey) and Gondar region (white) in North-Western Ethiopia. The size of the circles is proportional to the number of isolates found

In total, twenty different *spa* types were found, the majority of isolates belonging to t042 (58%) and the closely related t15786 (11%) and the distribution of *spa* types was similar across the two regions (Fig. 1). The proportion of *lukM-lukF’* positive *S. aureus* in our sample was low (3/79; 4%). Seven isolates carried *pvl* (7/79; 9%) and *tst* was detected in eight isolates (8/79, 10%). All t14061 isolates (3/79, 4%) were positive for *tst*; *pvl* was present in all t355 (3/79, 4%) and t1376 (2/79, 3%) isolates. None of the isolates carried the *mecA* gene. Table 2 summarizes the *spa* typing results and presents the isolates positive for virulence genes.

**Table 2:** Genetic characterization of 79 *Staphylococcus aureus* isolates from quarter milk samples of 60 cows in 42 dairy farms in North-Western Ethiopia

*spa* type	Total (%)	N isolates positive for			Repeat sequence										
		*lukM-lukF’*	*tst*	*pvl*											
t042	46 (58)	0	1	0	26	23	12					34	34	33	34
t15786	9 (11)	0	0	0	26	23	12				34	34	34	33	34
t355	3 (4)	0	0	3	07	56	12	17	16	16	33	31	57	12	
t14061	3 (4)	2	3	0	04	31	17								
t1376	2 (3)	0	0	2	07	12	21	17	13	13	34	34	33	13	
t488	2 (3)	0	0	0	07	12	21	17	13	13			34	33	34
t10018	1 (1)	1	1	0	04	31	17	25	17	25	17				
t306	1 (1)	0	1	0	26	23	17	34	17	20	17	12	17	17	16
t17835	1 (1)	0	1	1	14	22	75	16	24	24	24	24	24		
t223	1 (1)	0	1	0	26	23	13	23	05	17	25	17	25	16	28
t273	1 (1)	0	0	1	07	23	21	17	13		34	16	34	33	13
t2085	1 (1)	0	0	0	26									33	34
t4206	1 (1)	0	0	0	26	23						34	34	33	34
t9300	1 (1)	0	0	0	26	23	21								34
t4701	1 (1)	0	0	0	26	12	21	17			34	34	34	33	34
t17184	1 (1)	0	0	0	26	23	13			17	25	17			28
t2801	1 (1)	0	0	0	07	23	34	12	12	23	02	12	23		
t17834	1 (1)	0	0	0	07	23	02	12	23	02	02	02			34
t17185	1 (1)	0	0	0	07	500	22	31	17						
t409	1 (1)	0	0	0	60	61	34	22	34	17					

All six t042 isolates subjected to MLST typing belonged to a new ST, ST4550 which is a double locus variant of ST97. The other isolates subjected to MLST were ST1 (t273), ST22 (t17184), ST88 (t488) and ST848 (t409). All the data in this paper are available in Additional file 1.

### Antimicrobial resistance

All 79 isolates were susceptible for cefoxitin and were therefore classified as methicillin-susceptible *S. aureus*. Resistance to penicillin/ampicillin (86%) and tetracycline (54%) was high, while the resistance to other antimicrobials was limited. The MIC values and numbers of isolates resistant to the antimicrobials included in the testing are presented in Table 3. Associations between *spa*-type and AMR patterns were not found. Percentages resistance to antimicrobials by *spa*-type for 79 *S. aureus* isolates is presented in Additional file 2.

**Table 3:**
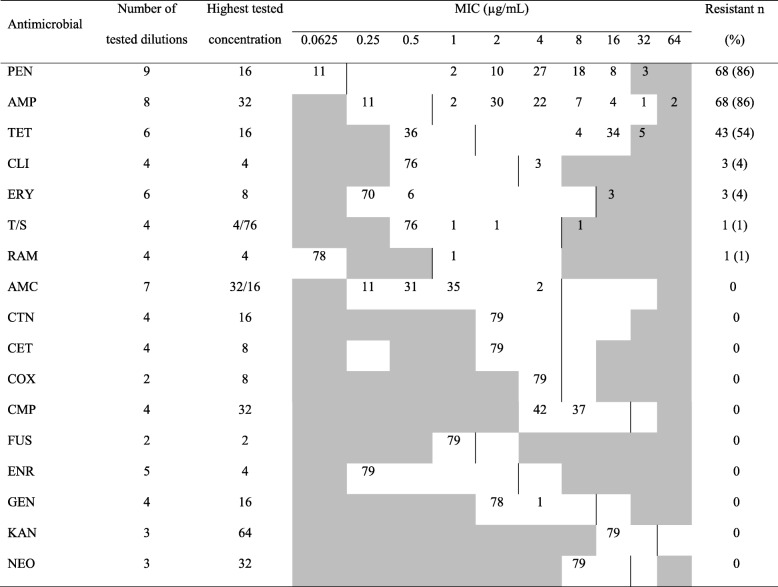
Minimum inhibitory concentrations (MIC) for 79 *S. aureus* isolates from dairy farms in North-Western Ethiopia tested for susceptibility to 17 antimicrobials^1^. The dilution ranges tested are those contained in the white area. Values above this range indicate MICs higher than the highest concentration within the tested range. Vertical lines indicate clinical breakpoints, with values to the left of the line being susceptible or intermediate and those to the right being resistant

^1^*PEN* penicillin, *AMP* ampicillin, *TET* tetracycline, *CLI* clindamycin, *ERY* erythromycin, *T/S* Trimethoprim sulfamethoxazole, *RAM* rifampicin, *AMC* amoxicillin/clavulanic acid, *CTN* cephalothin (1st generation cephalosporin), *CET* ceftiofur (3rd generation cephalosporin), *COX* cefoxitin, *CMP* chloramphenicol, *FUS* fusidic acid, *ENR* enrofloxacin, *GEN* gentamicin, *KAN* kanamycin, *NEO* neomycin

## Discussion

The aim of this study was to characterize *S. aureus* isolates cultured from milk samples of dairy farms in North-Western Ethiopia based on *spa* typing, virulence genes identification and AMR. The spa types t042 and the related t15786 were dominant, together representing 69% of the isolates. Such a high degree of relatedness suggests contagious transmission [13, 14]. Since our isolates were derived from different farms, contagious transmission is likely to occur not only within, but also between farms, possibly through cattle movements between farms, or from the government breeding and heifer distribution centers. Four of the *spa* types (t042, t223, t306 and t355) identified in the current study were previously isolated by Tarekgne et al. [9] in cattle in the Tigray region, Northern Ethiopia. Interestingly, in that study the dominant *spa* type was t314, which has a repeat sequence entirely different from the dominant types in our study. This suggests that, although contagious transmission seems to occur at a local scale, exchange of strains between regions seems to occur less, resulting in dominance of different clones in different regions. The Tigray region has a different cross-breeding and heifer distribution center, possibly explaining different dominant clones between regions. The six t042 isolates subjected to MLST belonged to the new ST4550, which is a double locus variant of ST97, a ST commonly found in bovine mastitis worldwide [30–32].

The proportion of isolates positive for *lukM-lukF’* genes was low. As the majority of isolates in our study belonged to a ST related to ST97, this low *lukM-lukF’* prevalence is in line with previous reports that found ST97 isolates to be negative for *lukM-lukF’* [16, 17]. The isolates that were positive for *lukM-lukF’* belonged to t14061 and t10018, the latter of which has previously been found in nasal swabs from Nigerian abattoir workers [33]. The bi-component leukocidin LukM-LukF’ is predominantly produced by strains isolated from bovine *S. aureus* mastitis [34] and specifically kills bovine neutrophils, whereas human neutrophils are not affected [18]. Based on an experimental study, Vrieling et al. [19] reported that LukM-LukF’ is produced during the course of infection and that a high levels in milk were associated with increased severity of mastitis. The low proportion of *lukM-lukF’* positive isolates in our samples may be partly due to the fact that they came from a cross-sectional study that included only two cases of clinical mastitis [35]. In our collection the proportion of isolates positive for *pvl* was higher than that of *lukM-lukF’*, despite the fact that bovine neutrophils are insensitive to this human-associated leukocidin [21]. Although there are reports of *pvl*-positive cattle *S. aureus* [16, 35], *pvl* is mainly detected in *S. aureus* of human origin [19, 36, 37] whereas cattle isolates are often negative for *pvl* [38–40]. The finding of *pvl* in isolates cultured from cows may suggest human to cow transmission of *S. aureus*. Schmidt et al. [41] reported transmission of a human ST8 isolate to a dairy cow which subsequently caused mastitis and speculated that the close contact between dairy cows and humans, especially in the milking parlor, enables such transmission. Under the Ethiopian circumstances, where cows are hand milked, such transmission seems to be even more likely. Further characterization of the *pvl*-positive *S. aureus* in this study and of *S. aureus* isolates cultured from farmers and their families may help quantifying the importance of human to cattle transmission of *S. aureus*. Likewise, similar studies on bovine isolates that may be found in humans may also show sharing of *S. aureus* isolates between cattle and their farmers, which likely occurs along the same transmission routes. The *tst* gene encodes the toxic shock syndrome toxin, which *S. aureus* uses to facilitate colonization [42]. Although the *tst* gene is documented to be more prevalent in bovine than in human *S. aureus* strains [43], we only found it in a low number of isolates.

In line with the report of Tigabu et al. [44] from the Central highlands of Ethiopia, we did not find *mecA* positive *S. aureus* in our sample. The majority of our isolates were, however, resistant to penicillin, ampicillin and tetracycline. Resistance to penicillin and ampicillin is common among mastitis causing *S. aureus*, but varies largely between geographical regions [45–47]. In Ethiopia, penicillin and ampicillin are commonly used antimicrobials to treat mastitis, and tetracycline is often used to treat other bacterial infections. The high level of resistance to these antimicrobials is in line with previous Ethiopian reports on isolates from bovine mastitis [6, 7, 48]. This consistent picture of widespread AMR in *S. aureus* is alarming and likely results from the high usage of antimicrobials, possibly due to the fact that farmers can obtain antimicrobials over the counter without a prescription. Additionally, farmers are often not instructed on a proper treatment regimen, because guidance by veterinarians is lacking. A final reason for the high level of AMR may be that Ethiopian farmers are not inclined to cull animals, but rather continue treating their cows over and over again for an extended period of time. Treatment of cows with mastitis during lactation is an important component of a standard mastitis control program for contagious pathogens. Because AMR to penicillin and ampicillin is common, amoxicillin-clavulanic acid may be used for treatment of IMI without the need for tetracyclines, macrolides, lincosamides or third-generation cephalosporins. However, antimicrobial treatment is less than desirably effective in eliminating existing *S. aureus* infections [49] and treatment of intramammary infections with penicillin-resistant *S. aureus* strains generally results in a lower cure rate for treatment with either *β*-lactam or non-*β*-lactam antibiotics [50]. If cows do not respond favorably to treatment, separation or culling of the infected animals can be used to limit further transmission, but in Ethiopia, dairy cows are not easily culled because cross breed cows are very valuable for reproduction, even if they are chronically infected and have repeated CM cases. Therefore, encouraging dairy farmers to prevent transmission of *S. aureus*, rather than to rely on antimicrobial therapy is a preferable approach for mastitis control in North-Western Ethiopia. For instance, milking hygiene can be improved [8] and post milking teat disinfection [51] is not very expensive, is easy to perform, and is known to be very effective and can be practiced by Ethiopian dairy farmers. Implementation of these and other control measures will likely result in a lower prevalence of mastitis.

## Conclusions

We showed a high degree of similarity between the bovine *S. aureus* isolates in North-Western Ethiopia, suggesting contagious transmission within and between farms. Moreover, there was a high level of resistance to antimicrobials that are commonly used to treat mastitis in Ethiopia. Therefore, implementing effective preventive measures aiming to limit transmission, rather than to use antimicrobials to control mastitis, along with guidelines for prudent use of antimicrobials, is important to decrease AMR.

## Additional files

Additional file 1: Dataset supporting the conclusions of this article. (XLS 79 kb)

Additional file 2: Percentage resistance to antimicrobials by *spa*-type for 79 *S. aureus* isolates. (DOCX 21 kb)

## Abbreviations

AMC: Amoxicillin/clavulanic acid
AMP: Ampicillin
AMR: Antimicrobial resistance
CET: Ceftiofur
CLI: Clindamycin
CLSI: Clinical and laboratory standards institute
CM: Clinical mastitis
CMP: Chloramphenicol
CMT: California mastitis test
COX: Cefoxitin
CTN: Cephalothin
ENR: Enrofloxacin
ERY: Erythromycin
FUS: Fusidic acid
GEN: Gentamicin
IMI: Intramammary infections
KAN: Kanamycin
MIC: Minimum inhibitory concentration
MST: Minimum spanning tree
NEO: Neomycin
PEN: Penicillin
RAM: Rifampicin
ST: Sequence type
T/S: Trimethoprim sulfamethoxazole
TET: Tetracycline
