# Supplementary material for: Characterization of Staphylococcus aureus isolated from milk samples of dairy cows in small holder farms of North-Western Ethiopia
Source: BMC Vet Res. 2018 Aug 23;14:246. doi: 10.1186/s12917-018-1558-1 (PMC6107951; doi:10.1186/s12917-018-1558-1)
Supplement: Supplementary file 2 — Percentage resistance to antimicrobials by spa-type for 79 S. aureus isolates. (DOCX 21 kb) [file 12917_2018_1558_MOESM2_ESM.docx]

Additional file 2: Percentage resistance to antimicrobials by *spa*-type for 79 *S. aureus* isolates identified from milk samples. Percentages of 0 are not shown.

| *spa* type | N isolates | Percentage of isolates resistant to antimicrobial^2^ | | | | | | |
| --- | --- | --- | --- | --- | --- | --- | --- | --- |
|  |  | PEN^1^/AMP | CLI | FUS | RAM | ERY | TET | T/S |
| t042 | 46 | 78 |  |  |  |  | 50 | 2 |
| t15786 | 9 | 100 | 11 |  |  | 11 | 44 |  |
| t14061 | 3 | 100 | 33 |  |  | 33 | 66 |  |
| t355 | 3 | 100 |  |  |  |  |  |  |
| t488 | 2 | 100 |  |  | 50 |  | 50 |  |
| t1376 | 2 | 100 |  |  |  |  | 50 |  |
| t10018 | 1 | 100 |  |  |  |  |  |  |
| t2085 | 1 |  |  |  |  |  |  |  |
| t223 | 1 | 100 | 100 |  |  | 100 | 100 |  |
| t273 | 1 | 100 |  |  |  |  |  |  |
| t4701 | 1 | 100 |  |  |  |  |  |  |
| t17184 | 1 | 100 |  | 100 |  |  | 100 |  |
| Other^3^ | 8 | 100 |  |  |  |  | 100 |  |

^1^ PEN=penicillin, AMP=ampicillin, CLI=clindamycin, FUS=fusidic acid, RAM=rifampicin, ERY=erythromycin, TET=tetracycline, T/S=Trimethoprim sulfamethoxazole.

^2^ Amoxicillin/clavulanic acid, cephalotin (1^st^ generation cephalosporin), ceftiofur (3^rd^ generation cephalosporin), cefoxitin, chloramphenicol, enrofloxacin, gentamicin, kanamycin, neomycin were also tested but are not given in the table as all isolates were susceptible to these antimicrobials.

^3^ Other spa types: *spa* types t2801, t9300, t17185, t306, t409, t4206, t17384, 17385.
